# Formulating Initial Programme Theories of the Healthy Homes and Neighbourhoods Integrated Care Initiative

**DOI:** 10.5334/ijic.6421

**Published:** 2022-11-21

**Authors:** John G. Eastwood, Ferdinand C. Mukumbang, Denise De Souza, Hueiming Liu, Erin Miller

**Affiliations:** 1 Sydney Local Health District, NSW Health, AU; 2 Sydney Institute for Women, Children and their Families, AU; 3 Ingham Institute for Applied Medical Research, Liverpool, NSW, AU; 4 School of Public Health and Community Medicine, University of New South Wales, AU; 5 Menzies Centre for Health Policy, The University of Sydney, AU; 6 Department of Global Health, School of Public Health, University of Washington, Seattle, WA, USA; 7 Torrens University, AU; 8 The George Institute for Global Health, University of New South Wales, AU

**Keywords:** critical realist evaluation, integrated care initiatives, programme theories, mechanisms

## Abstract

**Introduction:**

The Healthy Homes and Neighbourhoods (HHAN) integrated care initiative was designed to break intergenerational cycles of social and health inequalities and enhance access to and engagement with health and social services for vulnerable families in the Sydney Local Health District. We sought to unearth the initial programme theory of the HHAN initiative to inform rollout to other relevant areas.

**Methods:**

We conducted a critical realist evaluation using steps. (1) Exploring the events around the HHAN initiative development. (2) Explore consumer experiences. (3) Identifying the entities and associations characterising the HHAN initiative and related outcomes. (4) Searching for different theoretical perspectives and explanations (abduction). (5) Hypothesising the mechanisms and [context] conditions that might have activated the generation of the HHAN outcomes (retroduction).

**Results:**

We identified three central mechanisms; trust, buy-in and motivation, and understanding family dynamics operating across consumer, provider and systems levels of the HHAN initiative.

**Discussion:**

These programme theories reveal that to achieve the goals of HHAN, interpersonal dynamics, fostering buy-in and ensuring motivation of both the consumers and care workers should be sought and sustained at all levels.

**Conclusion:**

The programme theories unveil that integrated care initiatives should foster positive relationships at all levels to ensure favourable consumer outcomes.

## Introduction

Despite existing government initiatives designed to address the social, health and well-being inequities of Australian children and families who live in disadvantaged communities, inequality continues to grow and subsequently transferred to the next generation [1]. Consequently, underprivileged families find themselves in intergenerational cycles of poverty, violence and crime, poor education, limited employment opportunities, poor lifestyles, and unhealthy behaviours. These families become vulnerable to adverse social, physical, and mental health issues, which may remain hidden from social and health service providers and policymakers. Tackling such social determinants of health requires innovative integrated health and social care initiatives. An integrated care initiative is “a coherent set of methods and models on the funding, administrative, organisational, service delivery and clinical levels designed to create connectivity, alignment and collaboration within and between the cure and care sectors” [2]. The goal of these methods and models is to “enhance the quality of care and quality of life, consumer satisfaction and system efficiency for people by cutting across multiple services, providers and settings” [3].

In July 2015, the Sydney Local Health District (SLHD) implemented an integrated care initiative, the Healthy Homes, and Neighbourhoods (HHAN), for vulnerable families in the inner West of Sydney, Australia. The HHAN initiative was designed as a population-based and family-centred care-coordination network operating across agencies to assist vulnerable families in navigating the health and social care system [4]. The HHAN Integrated Care initiative seeks to (1) break intergenerational cycles of social and health inequalities, (2) enhance vulnerable families’ access to and engagement with health and social service, and (3) improve the care of families with complex health and social needs in SLHD. The HHAN initiative design used the following approach:

- Multiple core and non-core agencies work with vulnerable families over a sustained period (5 years).
- Co-design and co-production of the initiative in partnership with families and service partners
- All the needs of enrolled families are in the scope of the intervention, including housing, employment, income support and legal advice
- An early intervention and public health approach to interrupting cycles of family disadvantage, poor health, and psychological trauma
- Efficient use and leveraging from existing family, societal and government resources, including Medicare scheduled services
- Use of evidence-informed integrated care methods by service partners, including family case conferencing, and wrap-around care delivery
- Encouraging families to have a ‘health home’ for all their health needs and supporting progress toward self-efficacy
- Providing a supporting structure for general practice providers to care for families is often considered ‘too difficult.’
- Development and implementation of shared assessment tools and referral criteria
- Implementing family assessment and engagement tools to monitor the health and well-being of family members” [5].

For the HHAN initiative to be successful, the original design embedded consumer-centeredness, participation, and care coordination as its central tenets. To this end, the HHAN initiative includes the following intervention modalities – activities: identification of vulnerable family cohorts; care coordination; evidence-informed intervention(s); general practice engagement and support; family health improvement; placed-based neighbourhood initiatives; interagency system change and collaborative planning; monitoring of individual and family outcomes; and evaluation [6]. The implementation of HHAN in inner western Sydney initially had two place-based initiatives (PBI) in areas of heightened disadvantage providing integrated housing, drug and alcohol services, and financial and legal services. This integration approach was adopted to locally transform care delivery to improve patient health [and well-being] outcomes and reduce costs emanating from the inappropriate implementation of care across hospital and primary care services [7].

A preliminary big data outcome-based evaluation of the pilot HHAN initiative indicated a significant reduction in potentially preventable hospitalisation, emergency department visits, admissions and length of stay for members of HHAN families [4]. The evaluation further revealed that these reductions were more significant in the second year after family enrolment in the HHAN initiative [4]. While integrated services are considered beneficial over streamlined and focused services, evaluating their direct impact on informing rollout and policymaking remains challenging. Consequently, theory-driven approaches to evaluation, such as critical realist-informed evaluation, have been proposed as a suitable approach for evaluating complex interventions in open systems [8,9].

The translation research design of HHAN adopted the four phases of the 2008 Medical Research Council Framework [10] for the evaluation of complex interventions: development, feasibility/piloting, evaluation, and implementation. Theory-driven programme theories have been developed and modified using this cyclical framework at each phase. Initially, a Theory of Change logic model [7] and a critical realist-informed approach were used to construct folks’ theories – ancillary background theories [11] from programme designers’ perspectives informing the design of the HHAN initiative [7]. After piloting the HHAN initiative, critical realist evaluations were conducted to test the realist-informed folks’ theories [12]. Based on evidence obtained from these studies and other sources of information, we sought to formulate a refined initial programme theory. The initial programme theory is a set of explicit or implicit assumptions of how the programme is organised and why the programme is expected to work [13,14]. The initial programme theory of the HHAN initiative can be used to inform current and future policy for its further implementation and regional rollout.

## Theory and methods

### Methodological approach

We adopted a critical realist theoretical paradigm to generate the initial programme theories of the HHAN initiative. The initial programme theories were based partly on the realist theory and propositions of neighbourhood, context, stress, depression and developmental origins of health and disease [15]. The value of a realist perspective for designing and evaluating integrated care initiatives has been highlighted [16]. Critical realist approaches have value in improving evidence-based practice because they can disentangle the intricacies around the design, implementation and uptake of integrated care initiatives while retaining the notion of complexity that exists in their practical implementation.

Critical realism focuses on developing theories and models to explain social events, including how and why programmes work or not. According to McEvoy and Richards [17], critical realism provides a coherent framework for evaluation research based on our understanding causal mechanisms. Critical realist-informed research, therefore, adopts a theoretical approach, which deals with conceptualising the possible ways social entities act to produce possible outcomes. This approach offers the advantage that HHAN-related outcomes could be explained by studying the mechanisms that produce them, following the conditions introduced by the HHAN initiative into the context under investigation.

Critical realism posits that reality comprises the domains of the Empirical – experienced and observed events; Actual – events which occur but may or may not be observed/experienced; and Real – structures and mechanisms with the potential to produce events under certain conditions [18]. In realism, reality constitutes an observable and unobservable deep dimension (the level of generative mechanisms). The role of realist-informed research is to uncover the entities operating at the unobservable deep [19].

Central to critical realist methodology is the identifying and conceptualising of mechanisms and structures considered as the causal elements of events [20]. A multi-layered understanding of reality incorporates investigating different mechanisms operating at the psychological level of self, the level of situated activity, and the levels of intermediate and macro-level services. These mechanisms cannot be seen operating directly but can be inferred through a combination of empirical investigations [17]. Mechanisms alone do not explain how observed events occur. Critical realists emphasise that the effects of mechanisms are contextual – dependent on other structures and contextual elements with generative powers. The implication is that a mechanism does not always produce the same outcome in different contexts, a notion described as *contingent causality* [21].

Owing to the multi-component nature of the HHAN initiative, we found it helpful to adopt the Context-Intervention-Mechanism-Outcome (CIMO) heuristic tool to develop the initial programme theories of the HHAN initiative. Based on the critical realist understanding of causality, mechanisms (M), context (C), and outcomes (O) are predominantly used to express how and why programmes work or fail to work [21]. Other authors find it helpful to add the intervention element to their explanatory model to uncover how the different programme modalities work to achieve the intended outcome(s). In this way, the explanatory theory or model focuses on how and why the intended outcome (O) occurs because of the intervention (I) or its components introducing and triggering underlying mechanism(s) (M), operating in contexts (C).

### Methods

**Figure 1:**
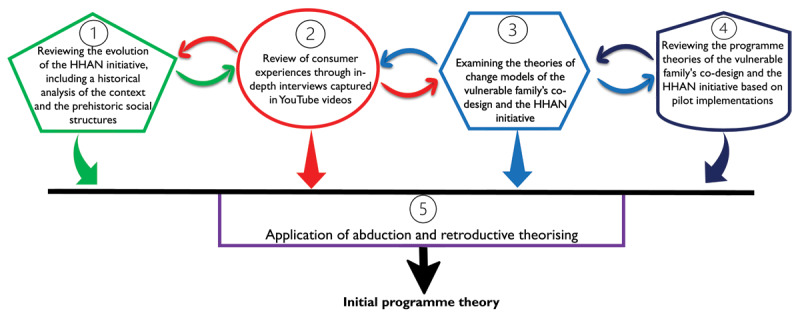
Iterative process toward developing the initial programme theories.

Our overall methodology followed guidelines proposed by Thapa and Omland [22]: (1) Exploring the events around the HHAN initiative development. (2) Identifying the entities and associations characterising the HHAN initiative and related outcomes and collecting data about these entities. (3) Searching for different theoretical perspectives and different explanations (abduction). (4) Hypothesising the mechanisms and conditions that might have activated the generation of the HHAN outcomes (retroduction). We followed the following steps in line with the principles outlined above:

The steps described above followed an iterative process indicated in Figure 1.

#### Step 1: Reviewing the evolution of the HHAN initiative

The review of the development of the HHAN initiative was achieved by thoroughly examining relevant policy documents, implementation documents, and notes. Vital documents that were consulted include:

1. SLHD Plans: https://www.slhd.nsw.gov.au/sydneyconnect/plans.html particularly the Community Health Services Strategic Plan, Inner West Sydney Youth Health and Wellbeing Plan, District Strategic Plan 2018–2023, and Child Health and Wellbeing Plan 2016–2021.
2. NSW Ministry of Health Vulnerable Families Transformation Plan: This document is the implementation plan that the Ministry wrote, based on the HHAN model.
3. Inner West Sydney: Healthy Homes and Neighbourhoods – 2015–2016 Annual Report https://www.slhd.nsw.gov.au/pdfs/HealthyHomesAnnualReport.pdf.

In addition to the review of the documents, zoom meetings were held with some stakeholders, including medical practitioners (4), researchers (6) and programme designers (3) working at the SLHD to discuss how the HHAN initiative was designed to achieve its aims. Monthly zoom meetings were conducted for three months to unpack the aims and objectives of the HHAN initiative. The goal of these zoom meetings was to assess the initiative’s progress, plan to address the barriers to implementation, and enhance the implementation of what is working well. These zoom sessions were recorded with permission from all participants. Meeting notes and summaries were prepared after each meeting and analysed thematically.

We meticulously read through the HHAN initiative documents, and in tandem with the meeting discussions, we abductively started formulating the programme theories of the HHAN initiative. Abduction at this stage was applied to generate hypotheses about the possible generative mechanisms that might operate, given the structure of the HHAN initiative and how it was designed to work.

#### Step 2: Exploring the experiences of consumer users

YouTube videos of HHAN care coordination consumers and their journey with HHAN were also used to substantiate the evidence obtained from the other sources. For ethical reasons, these videos will not be made publicly available. They were created by SLHD Strategic Relations and Communications for the SLHD Innovations Symposium. These stories were selected as they demonstrated “care in the community”, which was the symposium’s theme. Making these videos allowed families to share their experiences from their perspectives and homes where they received their care from HHAN. The families were then invited to the symposium as well.

These various sources of information, including the YouTube videos, were analysed thematically guided by the search of constructs that represent context conditions, generative mechanisms and reported behaviour or outcomes, especially in line with the different components offered by the HHAN intervention.

#### Step 3: Examining the theories of change of the HHAN initiative

The second step we adopted was critically examining the existing theories of change (ToC) of the vulnerable family co-design project and the developed and piloted HHAN initiative. This exercise aimed to identify the critical programmatic components of each model and the goals that these components strive to achieve in isolation and synergistically. Another essential element that examining the ToC aimed to delineate was the various activities (inputs), which usually require the cooperation and co-production of service providers and service users. Such interactions can highlight potential mechanisms of the HHAN initiative. The activities also shed light on some contextual elements, such as the availability of resources for integration-related activities.

**Figure 2:**
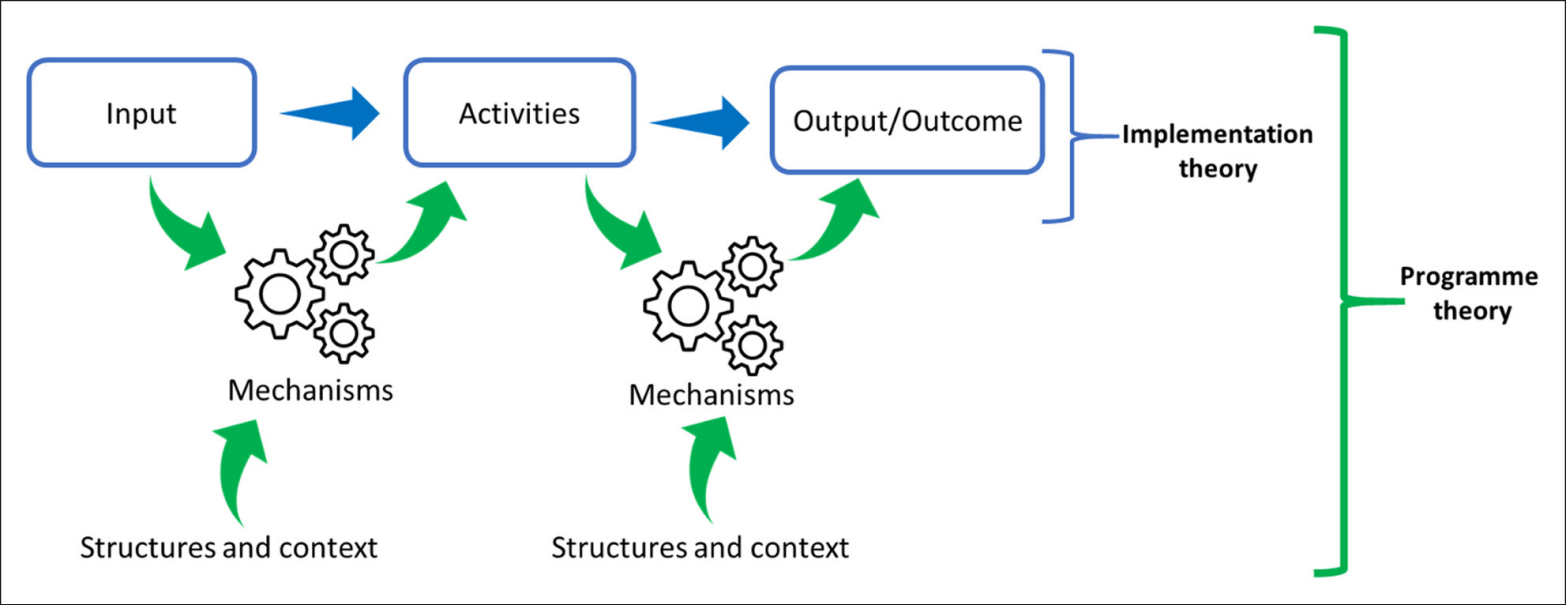
Potential information gleaned from exploring the theories of change (Adapted from Eastwood [7] and Kabongo et al. [23]).

Based on the framework presented in Figure 2, our goal was to initiate the process of identifying the different programme modalities, postulate potential mechanisms and identify the programme’s intended outcomes. This process also allowed us to link the context, intervention, mechanisms and outcome constructs abductively to shed some light on the initial programme theories of the HHAN initiative [24]. We were linking the elements of the CIMO heuristic tool involved abductively identifying the potential mechanisms and relating these to structures and context elements obtained from steps 1 and 2 through retroduction [25]—theorising the identified mechanisms to clarify how and why the HHAN initiative works or not and under what conditions (health systems, socio-economic and socio-political).

Following the review of the ToC, we delineated three levels of potential programme theories: consumer, provider, and service. While the consumer level speaks to the adoption of the HHAN initiative components by the families, the provider level relates to issues around the buy-in and consequent implementation of the HHAN initiative by the different providers, including health practitioners, case managers and social services providers. The programme theory at the service level deals with the coordination and organisation of separate independent systems to achieve integration. Table 1 illustrates the results of the exercises of this step.

**Table 1:**
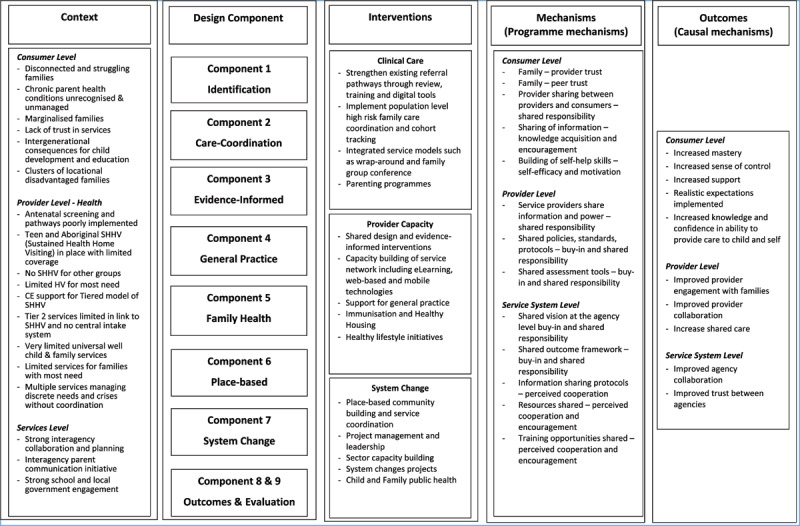
Illustrates the results of the exercises of this step.

#### Step 4: Reviewing available programme theories related to the HHAN initiative

Since the inception of the idea of developing HHAN, the critical realist approach to understanding how it will work, for whom and under what health system conditions has been considered [7,26]. The explanatory models were developed using the CIMO heuristic tool, which facilitated our theory development. Those efforts have been published in peer-reviewed journals [1,12,27,28,29]. This step examines these different explanatory models developed at the various stages of the HHAN initiative development. The goal was to revisit, confirm and revise the programme theories vis-à-vis information we obtained from reviewing the programme documents and examining the ToC from the preceding steps. Additional information to support the developing programme theories was obtained from other peer-reviewed articles on the programme descriptions, process evaluation and outcome-based evaluations of the HHAN initiative [4,15,26]. Articles reporting on place-based case studies were essential to understand what is working, for whom and in what circumstances within the neighbourhood integrated care initiatives.

The goal was to achieve theoretical constructs that would then be used to abductively and retroductively construct explanatory models of how and why the HHAN intervention works. Most importantly, this step allowed us to identify the other programme modalities and how they are operationalised at the consumer (user), provider and service levels. The findings of this step are presented in Tables 2, 3 and 4. Each table represents the programme theory from three different points of view: user/consumer, health care workers and provider and the operational/service provision levels.

**Table 2:** Consumer-level CIMO elements.


PROGRAMME MODALITY	CONTEXT	MECHANISM	OUTCOME

Accessibility	Consumer characteristics: – Vulnerability – (Dis)trust in health services Programme characteristics: – Home Visiting – Place-based initiatives Health system characteristics: – Inflexibility	– Family – peer trust – Provider sharing between providers and consumers – shared responsibility – Building of self-help skills – self-efficacy and motivation	– Consumers’ improved access to care – Service engagement

Referral pathways Early intervention and public health approaches to interrupting cycles of family disadvantage, poor health and psychological trauma	**Consumer characteristics:** – Vulnerability: Complexity of consumer’s problem – consumers’ past experiences with services and willingness to give the service provider a chance Programme characteristics: – Home Visiting – Place-based initiatives Health system characteristics: – Complex	– Sharing of information – knowledge acquisition and encouragement	– Effective engagement of consumers – Consumers’ improved access to care – Consumer outlook improved

Accompaniment Co-design and co-production of the initiative in partnership with families and service partners	Consumer characteristics: – Vulnerability – Disconnected from health services Programme characteristics: – Programme flexibility – Providers taking dual roles	– Provider sharing between providers and consumers – shared responsibility – Family–provider trust	– Consumer independence – Effective engagement of consumers – Consumers’ improved access to care – Consumer outlook improved

Case-based discussions: Encouraging families for all their health needs and supporting progress towards self-efficacy (I)	Consumer characteristics: – Vulnerability – Disconnected from health services Programme characteristics: – Programme flexibility – Providers taking dual roles	– Family – peer trust – Sharing between providers and consumers – shared responsibility – Sharing of information – knowledge acquisition and encouragement – Building of self-help skills – self-efficacy and motivation	– Effective engagement of consumers – Consumer priorities are reflected in initial goal setting


**Table 3:** Service provider-level CIMO elements.


CHARACTERISTIC OF PROGRAMME	CONTEXT	MECHANISM	OUTCOME

Family identification: Development and implementation of shared assessment tools and referral criteria	**Consumer characteristics:** – Level of trust between: – Consumer and index caseworker – Consumer’s family members and – caseworker – professionals involved (C) **Programme characteristics:** – Home Visiting – Place-based initiatives	– Multiple modes of communication – Perceived benefits of collaboration	Service engagement

Accompaniment/intensive hand holding	**Consumer characteristics:** – Vulnerability – Disconnected from health services **Programme characteristics:** – Programme flexibility – Providers taking dual roles	– Service providers share information and power – shared responsibility – Shared policies, standards, protocols – buy-in – Shared assessment tools – buy-in	Improved quality of service delivered

Evidence-based practice	**Consumer characteristics:** – Vulnerability – Diversity of age and social, cultural and health background **Programme characteristics:** – Flexibility	– Experienced clinicians – Leveraging pre-existing experiences – knowledge of local services – Experiential learning	– Improved quality of service delivered

Navigation of the health system	Health system characteristics: – Complex consumer characteristics – Poor health literacy – Distrust of health services	Experienced clinician – knowledge of local services – Experiential learning	Shared learning among professionals –Appropriate referrals


**Table 4:** Service organisation-level programme theory model.


CHARACTERISTIC OF PROGRAMME	CONTEXT	MECHANISM	OUTCOME

Service Collaboration	Health system characteristics: – Siloed health system – Resistant to cross-service collaboration – Lack of clarity about sharing consumer information Systemic barriers: – Socio-economic determinants of health – historical perceptions of health services – Poor health literacy – Geographical isolation from services Programme characteristics: – Complexity of the HHAN initiative makes it challenging to explain to other services Consumer characteristics: – Vulnerability	– Shared vision at the agency level buy-in and shared responsibility – Shared outcome framework – buy-in and shared responsibility – Information sharing protocols – perceived cooperation and trust – Resources shared – perceived cooperation and encouragement – Training opportunities shared – perceived cooperation and encouragement	– Breakdown of silos – Collaboration between services – Recognition by service partners – Utilising appropriate services – Acknowledgement and acceptance of HHAN – Foundations for integration


#### Step 5: Eliciting and refining the initial programme theories

This step is the culmination of the activities that we conducted from step 1 through step 4. This final step entailed the discursive reviewing of the developing initial programme theories from step 1. This step involved a discussion led by one of the authors with a group of researchers working on the HHAN intervention from SLHD since its inception and working on other integrated care programmes in Australia. This team of six was engaged in a discursive exercise reflecting on the developing programme models obtained from steps 1–3. Through these discussions, the causal links of the different CIMO chains were examined, and these chains were confirmed, discarded or revised. The exact process was also used to refine the developing programme theories.

## Results

We present here three programme theories aligning with the three levels: consumer, provider and service [30]. For each of these levels, we have provided a table illustrating the different elements of the CIMO heuristic framework. In addition, we constructed causality models using the constructs identified in the corresponding tables. Finally, we identified the central mechanisms operating across all three levels.

### The consumer-level

The programme theory developed from the consumers’ perspective is essential to understand the dynamics around the adoption of the programme. Of course, without the intended users adopting the intervention, the programme cannot achieve its goals. Table 2 illustrates the consumer-level CIMO elements that were distilled through the five steps of the research process.

**Figure 3:**
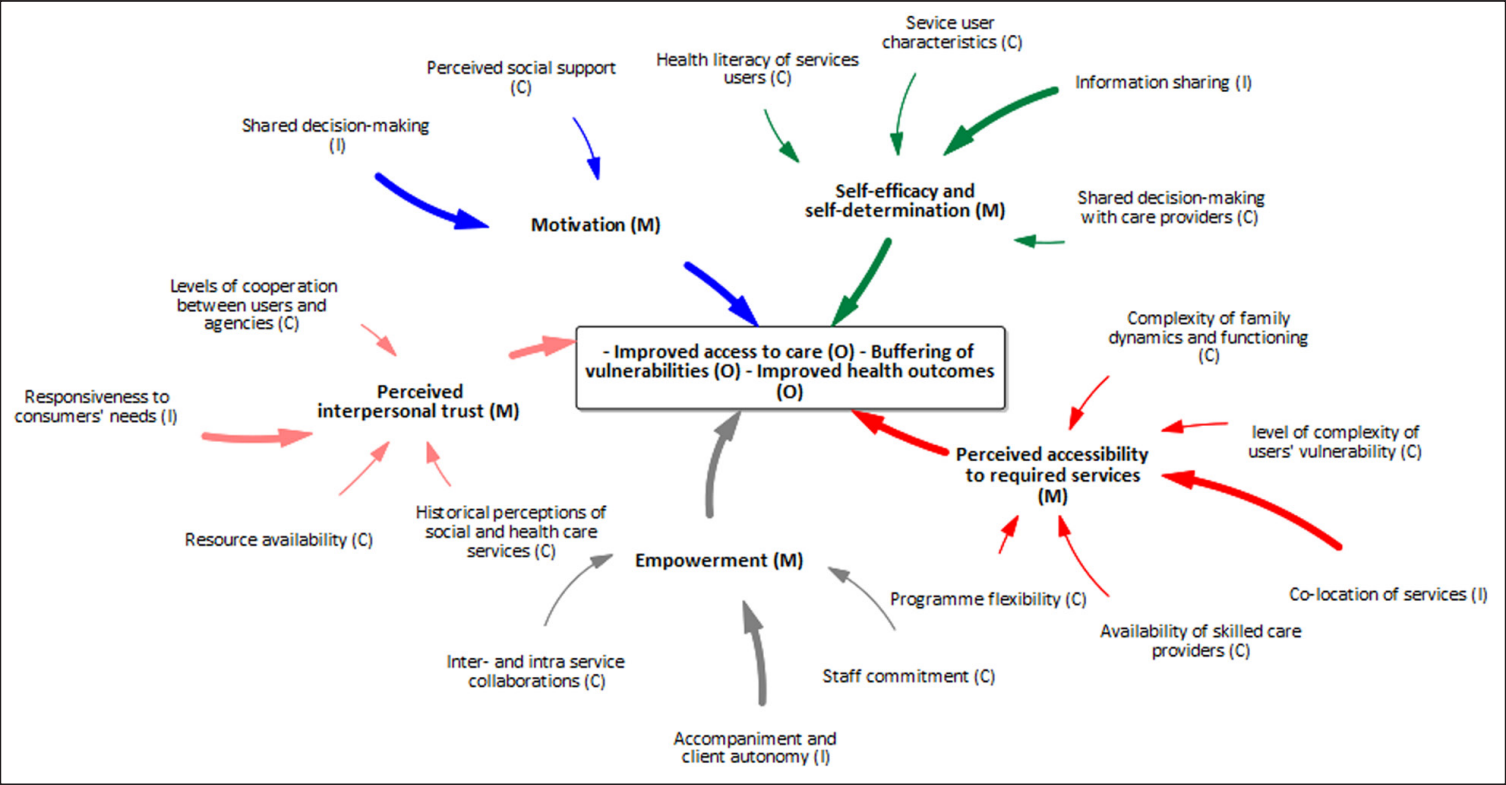
Consumer-level programme theory model.

Following the abductive and retroductive process, we constructed the programme theory model at the level of the HHAN initiative consumers – Figure 3.

The initial programme theory at the consumer level indicated that rapport building with all family members engenders mutual respect and trust (M) and encourages consumers to engage in their goal settings, consequently improving the uptake of social and health services. Case-based discussions fostered knowledge sharing and awareness of available activities leading to knowledge acquisition, encouragement, and empowerment. These three mechanisms have been found to promote positive outcomes such as consumer independence, an improved outlook, and community acceptance of the programme. Under contexts and conditions where vulnerable families experienced distrust of past health and social services—and low levels of trust in providers, authorities and the overall policy environment—a whole-of-family care approach can allow consumers to feel supported in their community.

#### Provider level

**Figure 4:**
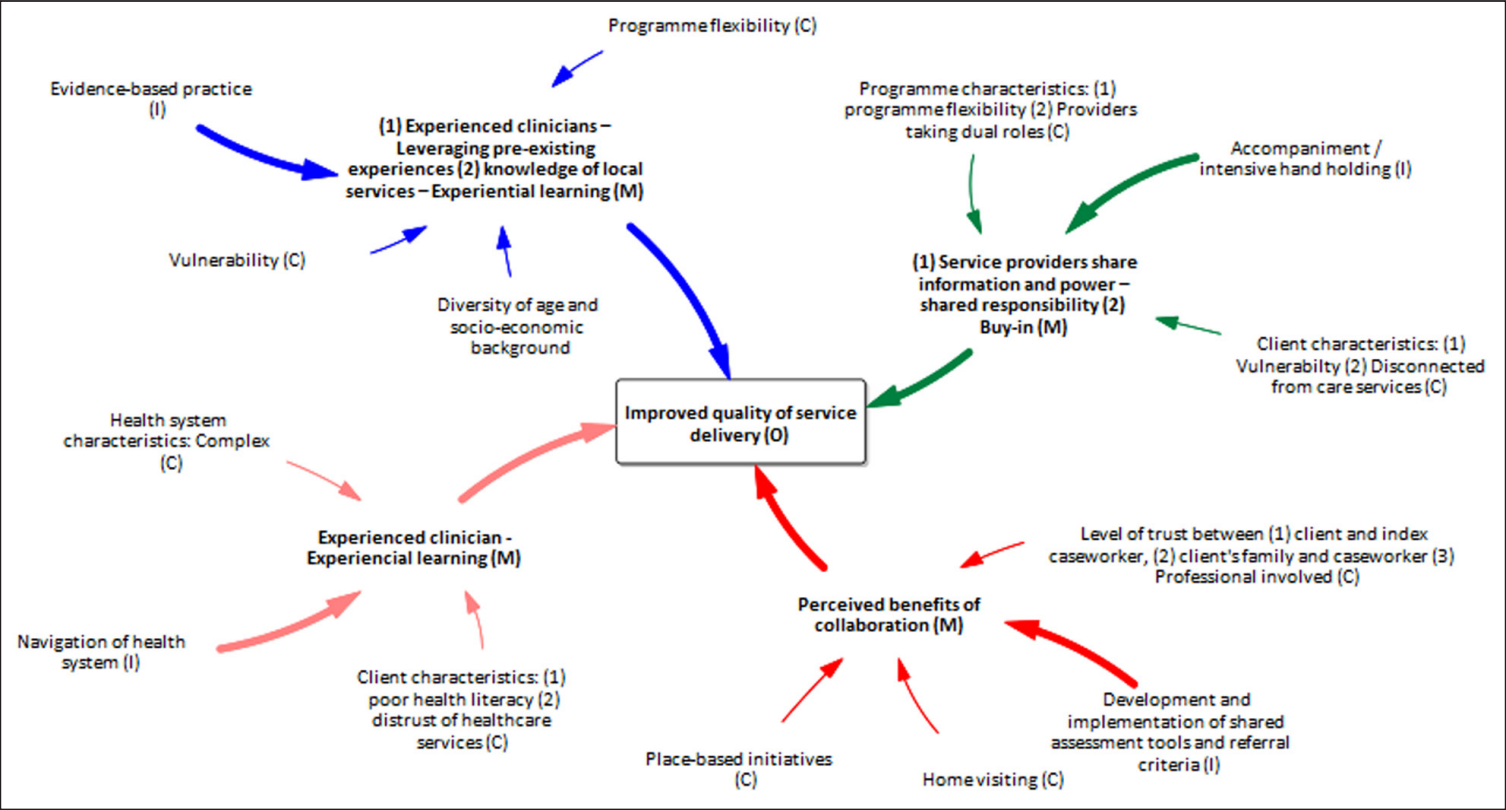
Service provider-level programme theory model.

The second programme theory we developed is at the provider level. This programme theory speaks to the implementation phase of the HHAN intervention as the success of the implementation of the HHAN intervention is mainly dependent on the care providers. Care providers are directly involved in delivering the HHAN intervention. Thus, they are considered the central actors vis-à-vis the implementation of the HHAN intervention. Table 3 illustrates the elements identified through the study efforts toward constructing the CIMO model (Figure 4).

#### Service organisation-level

**Figure 5:**
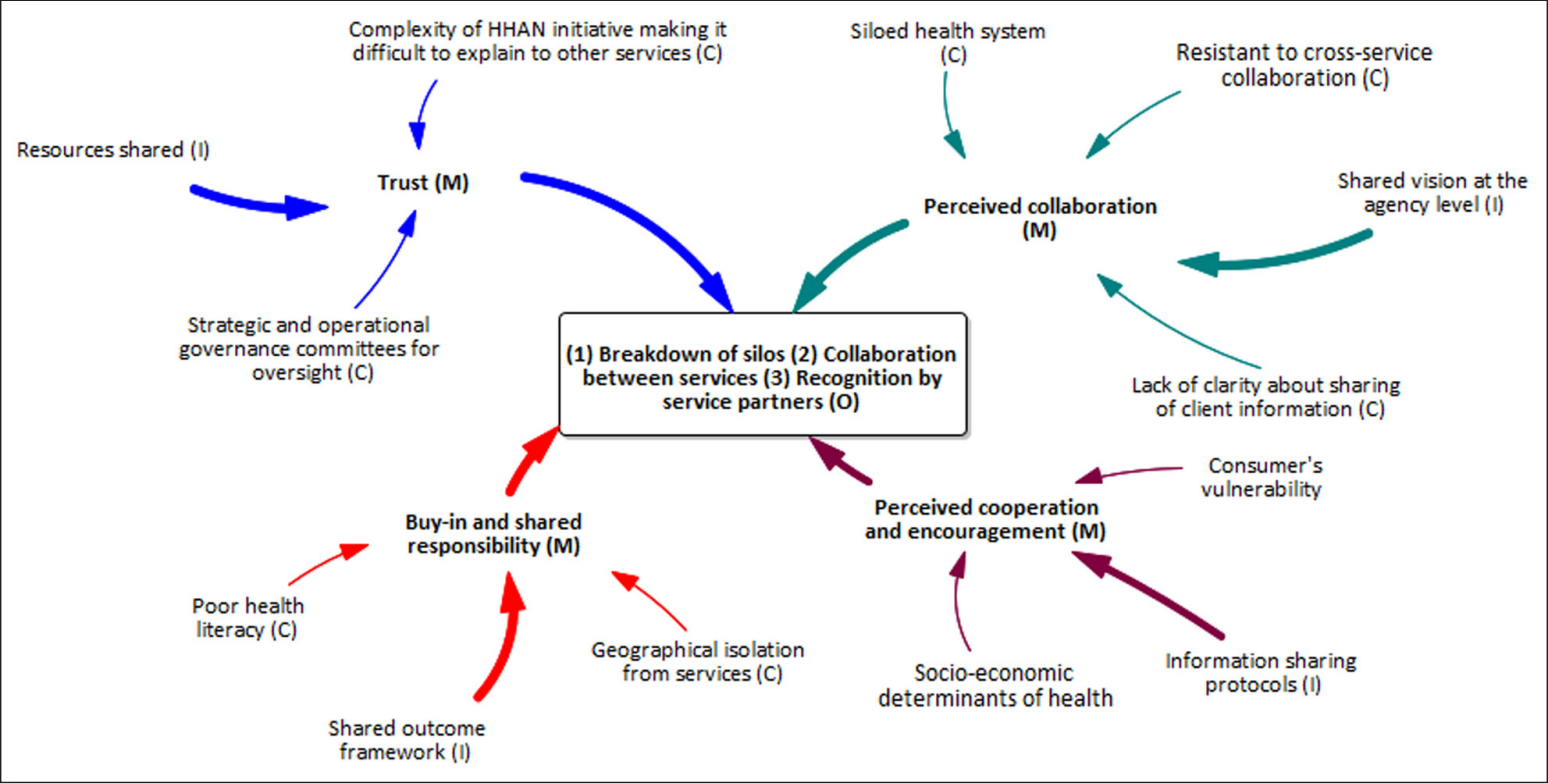
Service organisation-level programme theory model.

The service organisation-level deals with the interplay between policymakers and the rollout of the intervention at the implementation sites. This level highlights the complexity of how the integration of services is supposed to be achieved during implementation. Table 4 below illustrates the CIMO elements distilled towards constructing the related programme theory model (Figure 5).

#### Overarching mechanisms operating at all levels of HHAN

**Figure 6:**
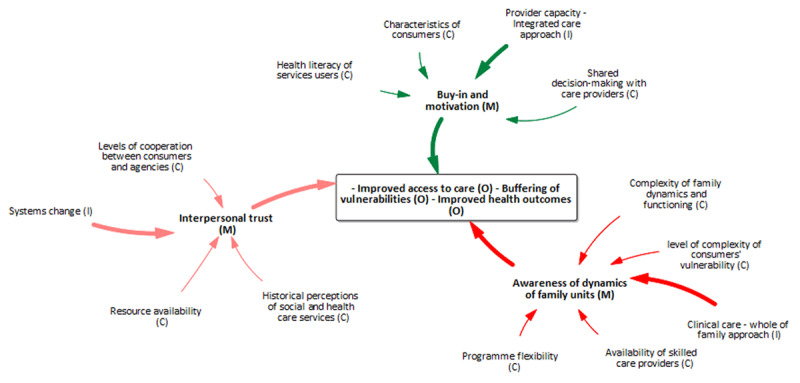
Overarching mechanisms present at all levels of the HHAN logic model.

Byng and colleagues [31] propose that while it is essential to have the CIMO configurations of the different levels of the programme, policy or intervention, it adds value to see how these units come together as a whole. To this end, we abductively identified three central mechanisms 1) interpersonal trust, 2) buy-in and motivation and 3) awareness of the dynamics of family units that operate at all three levels Figure 6. These three mechanisms also reveal the points of integrating the three levels of programme theories developed.

## Discussion

In this study, we employed a multi-method approach to elicit the initial programme theories of the HHAN initiative at three levels [32]. We formulated three programme theories for the levels of consumers, care providers and service systems. While the consumer-level programme theory is based on the adoption of the programme by vulnerable families, the care provider perspective focuses on the implementation of the intervention, and the services systems perspective deals with the rollout of the HHAN initiative. Although we have delineated these programme theories into three programme theories, they are intricately connected in the sense that what happens at one level affects what happens at the other levels. For example, poor implementation outcomes at the care provider level will affect the uptake or adoption of the intervention at the consumer level. This approach aligns with the complexity science approach, a theoretical approach to understanding interconnections among agents and how they give rise to emergent, dynamic, systems-level behaviours. Therefore, our adoption of this approach enables us to consider the dynamic properties of systems and the multiple forces, variables, and influences [33], which should be considered when designing and implementing an integrated care initiative such as HHAN.

Overall, trust—which emanates from favourable interpersonal relations—buy-in from the co-design and co-identification of problems and solutions, and motivation owing to knowledge acquisition were found to be overarching mechanisms, under which secondary modes of intervention at consumer, provider and system levels emerged. The centrality of trust, motivation and buy-in as enablers of service engagement and provider motivation to collaborate has also been highlighted in the recent literature [34,35,36]. These programme theories further illustrate that HHAN stakeholders—when implementing and rolling out the programme—should pay attention to ensuring the sustainability of interpersonal dynamics, fostering buy-in at all levels and ensuring motivation of both the consumers and care workers for the programme to be successful. To this end, service design should focus on fostering the creation of positive relationships at all levels to ensure good consumer outcomes.

We found that at the consumer level, critical mechanisms include awareness of family dynamics, knowledge acquisition, self-efficacy and encouragement embedded in trust. A recent realist review to identify causal mechanisms on how and why community-based integrated care initiatives work found five bundles of mechanisms at the consumer level (1) motivation, (2) perceived interpersonal trust, (3) user-empowerment, (4) perceived accessibility to required services, and (5) self-efficacy and self-determination [37]. These mechanisms align with those found in this review at the same level but for family dynamics. Trust was fundamental to the HHAN initiative’s functioning because it favours the formation of positive case worker-family unit bonds, which is central to the other modalities of the HHAN initiative, such as co-designing and co-production. Morton and Paice [38] found that co-producing and making decisions with consumers encouraged innovation, improved communication, and held the actions of other partners accountable to ensure the integrated care’s vision and goals were achieved. A systematic review conducted to uncover the underlying values of integrated care found other values, including co-designing and co-producing, as important elements of integrated care [39].

At the provider level, we found that if there is a good level of trust between care providers and the authorities, the presence of transformational leadership, and buy-in from the programme managers, shared learning among the professionals can be achieved, and the buy-in by staff and agency will increase. Thus, leading to perceived benefits of cooperation between professionals and agencies, experiential learning, mutual respect, and trust. To this end, while implementing or rolling out the HHAN intervention, strategies must be devised to improve stakeholder engagement and drive further collaboration. Evidence-based practice and family accompaniment, which meant working at multiple levels simultaneously and maximising partnership opportunities, activated shared and experiential learning mechanisms through multiagency shared casework. Leveraging learning from experiences and transferring learnings from the service level to the system level can enforce the transition from service collaboration to service integration. A realist evaluation of another integrated care initiative to improve the diagnoses and management of chronic conditions indicated that the initiative increased the confidence of the primary care professionals through improved person-centred conversational skills and increased patients’ active engagement [40].

In siloed health systems, strong interagency cooperation, high vulnerability, and the complex nature of the HHAN initiative, proper service integration can only be achieved if there is buy-in, shared responsibility, and perceived cooperation among the authorities and service providers. Smeets and colleagues [40] observed that mutual trust between primary care providers and patients and between primary care providers and their network partners improved the uptake of social and healthcare services among patients with chronic diseases. The above service provision-level theory highlights the need to pay further attention to the complexities of service collaboration within a healthcare context. Further, working at multiple levels simultaneously and maximising partnership opportunities, shared learning and knowledge transfer through multiagency shared casework may aid in transferring learnings from the service level to the system level and assist with the transition from service collaboration to service integration.

The notion of emergence captured in the principles of critical realism alludes those structures and mechanisms are usually unobservable, and their operations depend on situations with causal tendencies. Emergence occurs when a whole possesses one or more emergent properties. Within the systems in which the HHAN initiative operates, operations arise within and across different levels. The operations of mechanisms and structures at one level can have implications or create conditions for how the mechanisms operate at another level. In some instances, the outcome of one level constitutes the context conditions for the next level. For example, at the systems level, where outcomes such as the breakdown of silos, collaborations and recognition of service partners are achieved, can constitute favourable conditions for triggering the mechanisms at the service provider level. Therefore, the outcomes achieved at one level constitute situations with causal tendencies at the next level. Practically, this notion of emergence, as applied in this circumstance, suggests that relevant stakeholders should ensure that the outcomes at the higher levels are successfully achieved to create favourable conditions for attaining those at the lower levels.

While we attempted to capture the complexity around the implementation and uptake of the HHAN initiative, we also want to recognise that the continuous activation of essential mechanisms such as buy-in, trust and motivation, and acceptance for favourable outcomes are also contingent upon how things play out over time. This time consideration is in line with the temporospatial dynamics of events captured in the critical realist philosophy of science. In keeping with the notion that the world is changing and that social structures, mechanisms, and causes operate in a complex system [41], we appreciate that activated mechanisms such as those identified at the different levels are likely to change. For example, losing the momentum of motivation or trust over time as the other actors or service providers change through staff turnover or rotations. These personnel changes have implications for job design, recruitment, workforce capability development, and service-to-service communication pathways and should be factored into the timeline for programme implementation.

We propose, therefore, that structures should be set in place to ensure affective, continuance and normative commitments at all levels, with the relevant stakeholders operating at each level. Meaningful engagement can be achieved by assessing the programme’s success, addressing existing barriers, fostering existing relationships and encouraging active participation and co-decision-making (service providers – consumers) at all levels. Such engagements can be achieved through frequent meetings and re-evaluating the aim and commitment of the different service providers and stakeholders. Ensuring the resources for implementing various levels and aspects of the HHAN programme is also critical to sustaining buy-in, trust and motivation.

## Strengths and limitations

The study was conducted based on the critical realist philosophy of science, which offers a multilevel perspective fostering the investigation of different levels of mechanisms operating at the psychological level of self, the level of situated activity, and the levels of intermediate and macro-level services. This feature of the critical realist methodology allowed us to unearth the programme theories of the HHAN initiative at three levels: consumer, care provider and service provision.

Another strength of the critical realist-informed evaluation approach is that as opposed to outcome-based evaluations, where contextual elements are eliminated to establish the association between the programme modality and the observed outcome, retroductive theorising is inclusive of the contextual elements in its evidence package [42]. Obtaining evidence through examination of context provides practical knowledge for implementers to make contextual judgments about what is likely to work under what contextual conditions and for what section of the population. Because critical realist-informed studies apply retroduction, they have the potential to provide context-linked evidence to increase adoption and usability.

The study, however, has some limitations. Constructing CIMO configurations for each significant element requires abductive thinking and retroduction to link the different elements, which makes replication of such studies challenging, requiring a high level of expertise. Although we engaged in discursive and iterative discussions, the risk of misattribution of causality remained, primarily because the study is conducted in an open system within a multi-component programme. In our comprehensive methods, we included respondent feedback/discussions with the programme architect and team to revise and confirm our causal links to minimise such misappropriation of causality. Several analytical methods were employed through theory gleaning, refining, and consolidating.

## Conclusion

We adopted a complex systems-sensitive approach to evaluate the HHAN integrated care initiative. Capturing the central mechanisms at work at each level using a causality-based evaluation approach helps us understand how mechanisms at one level can impact the implementation and adoption of the different components of HHAN integrated care at other levels. These programme theories revealed that to achieve the goals of the HHAN integrated care initiative, interpersonal dynamics, fostering buy-in at all levels and ensuring motivation of both the consumers and care workers should be sought during implementation. The programme theories unveil that integrated care service design should focus on fostering positive relationships at all levels to ensure good consumer outcomes, which could be achieved through scheduled collaborative meetings.

## Figures and Tables

**Table:** 

CIMO:	Context-Intervention-Mechanism-Outcome
SLHD:	Sydney Local Health District
HHAN:	Healthy Homes and Neighbourhoods
ToC:	Theory of Change
